# Appetitively motivated tasks in the IntelliCage reveal a higher motivational cost of spatial learning in male than female mice

**DOI:** 10.3389/fnbeh.2024.1270159

**Published:** 2024-02-29

**Authors:** Martina Nigri, Giulia Bramati, Adrian C. Steiner, David P. Wolfer

**Affiliations:** ^1^Department of Health Sciences and Technology, Institute of Human Movement Sciences and Sport, ETH Zurich, Zurich, Switzerland; ^2^Institute of Anatomy, Faculty of Medicine, University of Zürich, Zürich, Switzerland

**Keywords:** IntelliCage automated system, appetitive learning, 3R refinement, C57BL/6J mice, sex differences, animal welfare

## Abstract

The IntelliCage (IC) permits the assessment of the behavior and learning abilities of mice in a social home cage context. To overcome water deprivation as an aversive driver of learning, we developed protocols in which spatial learning is motivated appetitively by the preference of mice for sweetened over plain water. While plain water is available at all times, only correct task responses give access to sweetened water rewards. Under these conditions, C57BL/6J mice successfully mastered a corner preference task with the reversal and also learned a more difficult time-place task with reversal. However, the rate of responding to sweetened water decreased strongly with increasing task difficulty, indicating that learning challenges and reduced success in obtaining rewards decreased the motivation of the animals to seek sweetened water. While C57BL/6J mice of both sexes showed similar initial taste preferences and learned similarly well in simple learning tasks, the rate of responding to sweetened water and performance dropped more rapidly in male than in female mice in response to increasing learning challenges. Taken together, our data indicate that male mice can have a disadvantage relative to females in mastering difficult, appetitively motivated learning tasks, likely due to sex differences in value-based decision-making.

## Introduction

The behavioral characterization of wild-type and genetically modified mouse strains has become a powerful tool for investigating the molecular basis of normal brain functions (van der Staay et al., 2009; Voikar, 2020) and dysfunctions (Moore et al., 2021; Brilkova et al., 2022; Shcherbakov et al., 2022). In this context, most of the experimental studies conducted with rodents have traditionally used male subjects, very rarely offering adequate comparisons between males and females. This is likely associated with the assumption that females might display a larger variability due to the estrous cycle (Prendergast et al., 2014; Shansky, 2019). However, differences between the sexes have been documented at every level of neuroscience, from single neurons in cell culture to systems-level processes measured by neuroimaging (Andreano and Cahill, 2009; Arnold, 2010; Clayton and Collins, 2014; Meyer et al., 2017). The claim that these neurobiological sex differences extend to the behavioral level has typically been more controversial. Given that free-living male *Mus musculus* have larger territories and venture farther away than females (Pocock et al., 2004, 2005), one would predict sex differences in spatial learning and exploratory behavior in laboratory tests. However, while literature reports indicate large and reliable male advantages for rats in radial-maze and water-maze protocols (Jonasson, 2005), experimental findings have remained contradictory in laboratory mice (Frick et al., 1999; Võikar et al., 2001; Hendershott et al., 2016). For example, evidence suggests differential performance by male and female mice in spatial navigation tasks (Kundey et al., 2019) and object recognition tasks (Frick and Gresack, 2003). In line with these observations, experimental studies reported poorer performance in the water maze combined with increased serum corticosterone levels in females (Beiko et al., 2004). In contrast, the equivalent performance of female and male C57BL/6J mice in the open field and water-maze task have been reported in previous studies (Fritz et al., 2017). Sex differences can also emerge in decision-making where an animal is given a choice between an option that provides a smaller but guaranteed gain and an option that provides a larger gain but also could provide a loss. In humans, it is well-established that men tend to be more risk-seeking than women in a wide domain of decision-making (Fornwagner et al., 2022), gambling (Raylu and Oei, 2002; van den Bos et al., 2013a), and financial risk-taking (Dwyer et al., 2002; Eckel and Grossman, 2002; Charness and Gneezy, 2012). In contrast, studies on non-human animals, including common laboratory mice, have been limited in their conclusions.

Along with the widespread use of the behavioral phenotyping approach, a large variety of rodent behavioral tests has been established to evaluate various forms of cognitive functions (Morris, 1981; Pellow et al., 1985; Vorhees and Williams, 2006; Hånell and Marklund, 2014). Despite their efficacy, classical tests still must cope with a few limitations. In fact, traditional behavioral tests typically involve social isolation, sensory deprivation, exposure to unfamiliar apparatus with very short observation time, and repeated handling by humans. The resulting stress responses introduce artifacts and reduce test reliability (Crabbe et al., 1999; Chesler et al., 2002; Deacon, 2006; Endo et al., 2011; Voikar and Gaburro, 2020). In addition, an anxiety-inducing experimenter effect is always present (Nigri et al., 2022). These shortcomings have, therefore, created an urgent need to develop new, more efficient approaches to behavioral phenotyping of mice. Therefore, a number of computer-assisted technologies for automatically capturing rodent behavior in the home cage over long periods of time have been developed (Gerlai, 2002; Spruijt and Devisser, 2006; Goulding et al., 2008; Endo et al., 2011; Kahnau et al., 2023). Among them, the IntelliCage (IC) is a unique approach because the system is specifically designed for the cognitive assessment of group-housed mice. Advantages of such automated testing in the home cage compared to manual assessments include continuous monitoring, observation in a familiar environment, and examination of combinations of behaviors rather than single behaviors (Richter, 2020; d’Isa and Gerlai, 2023). Moreover, experimental paradigms and protocols can be freely programmed and executed with this system, thus allowing maximum flexibility in the experimental design. The automated generation and collection of data by standardized procedures allow for high data comparability and reproducibility among different laboratories. Additionally, the apparatus also minimizes the need for the experimenter’s handling, thus reducing the artifacts that interfere with the activities of the mice.

Even though the IntelliCage system offers the mentioned advantages, thirst remains the driver of learning and only correct responses grant access to drinking water in typical IntelliCage learning tasks. Thus, poor learning or insisting on wrong response patterns may result in water deprivation, which negatively impacts animal welfare. To refine the approach in accordance with the 3R principles (replace, reduce, and refine), we designed IntelliCage learning tasks in which successful learning gives access to a sweet reward while plain water is constantly available. In a previous study, we were able to show that this purely appetitive motivation is sufficient to drive the learning of female mice in simple IntelliCage tasks but fails in more complex hippocampus-dependent tasks (Bramati et al., 2023). This was achieved by exploiting the known preference for saccharin of C57BL/6J mice (Bachmanov et al., 2001). In the present study, we sought to determine whether using access to a saccharin reward as a sole and purely appetitive learning incentive could also be used to motivate male mice to learn simple IntelliCage tasks and whether they would lose interest in learning at a similar turning point as female mice if task difficulty is increased.

In the above-mentioned previous study (Bramati et al., 2023), the mice had the option to first respond to saccharin and switch to plain water during the same visit as a backup after not being rewarded with saccharin in an incorrect corner. The second aim of the present study was to test whether this option of double choices could have contributed to their rapid decline in performance as learning tasks became more difficult. To this end, we introduced a modified protocol enforcing an exclusive choice of either plain water or sweet water reward during each visit and compared it with the standard protocol used in the previous study. C57BL/6J mice, the most commonly used inbred strain in behavioral genetics, were deliberately chosen in both studies. For many behavioral domains, they are considered to display a medium-level phenotype (Crawley et al., 1997), which allows a feasible detection of upward and downward behavioral changes at the baseline and in response to various manipulations (Stiedl et al., 1999; Cabib et al., 2000; Võikar et al., 2005).

## Materials and methods

### Animals and environment

All the animal experiments were carried out at the Institute of Anatomy, University of Zurich, in accordance with the European legislation (Directive 2010/63/EU) and have been approved by the veterinary office of the Canton of Zurich (License number 060/2021).

**Figure 1:**
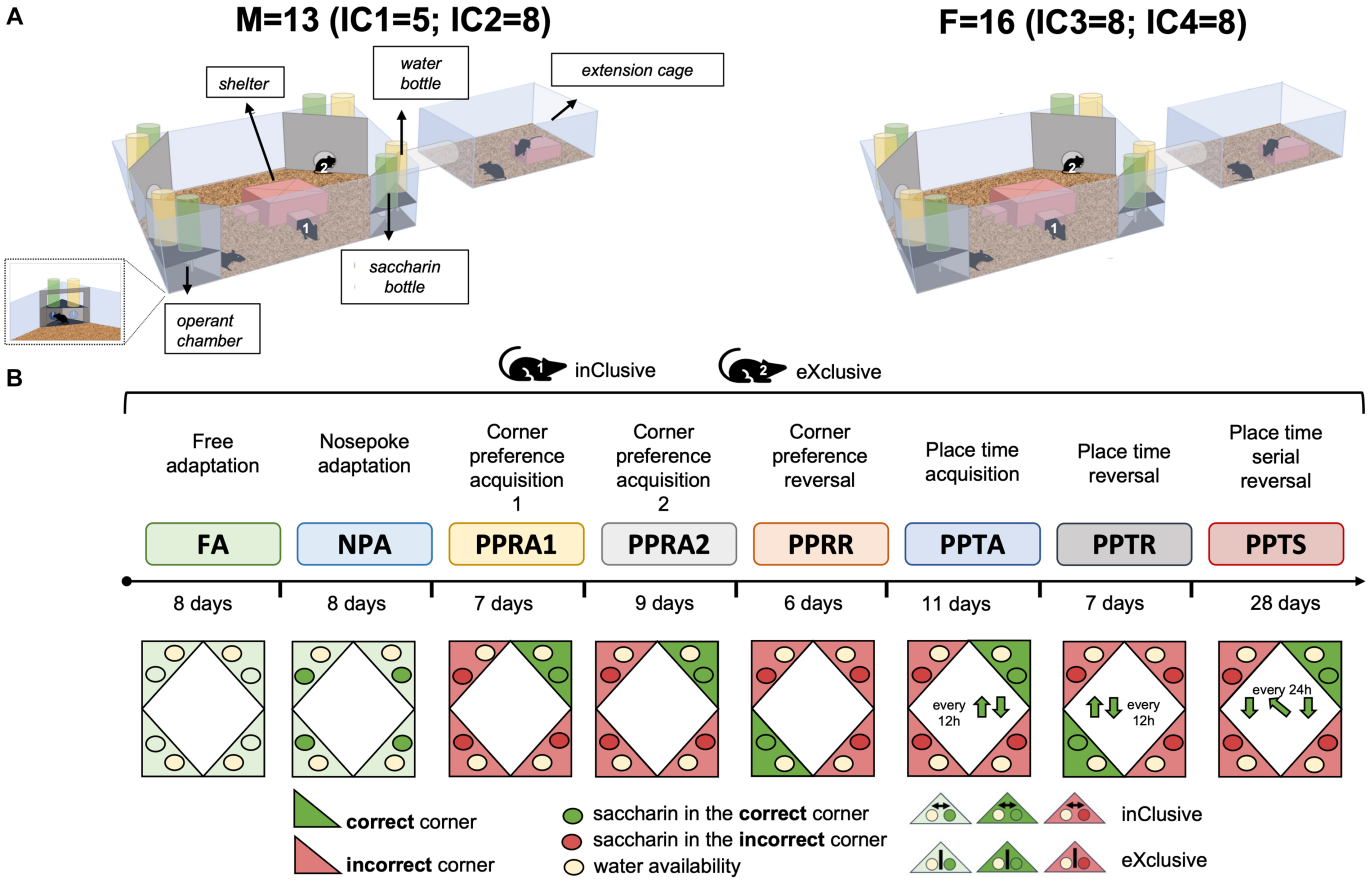
Apparatus and task paradigm. **(A)** Overview of the IntelliCage apparatus. C57BL/6J males (*N* = 13) and females (*N* = 16), assigned to two experimental groups, the exclusive choice group (*F* = 8, *M* = 6) and inclusive choice control group (*F* = 8, *M* = 7), were group-housed in the IntelliCage system (IC1, IC2, IC3, and IC4). While the IntelliCage 1 accommodated 5 male mice (inclusive choice = 3, exclusive choice = 2), the IntelliCage 2 accommodated 8 males (inclusive choice = 4, exclusive choice = 4). The IntelliCages 3 and 4 accommodated 8 female mice each (inclusive choice = 4, exclusive choice = 4). Their behavioral response, corner visits, nosepokes, and licks were monitored in a fully automated manner during the experimental tasks. **(B)** Diagram of behavioral test sequence based on appetitive learning.

Male and female C57BL/6J mice were bred at the Institute of Anatomy housing facility. Animals (*N* = 29, *F* = 16, *M* = 13) were weaned at 21 days and kept in the same-sex groups in standard Type III cages (temperature 21.9 ± 0.3°C and relative humidity 60.2 ± 9.6%) under a 12/12 inverted light-dark cycle (light on 20:00–08:00) for an adaptation period. A maximum of two pups per sex per litter were group-housed to avoid litter effects. Food and water were provided *ad libitum*. The radio frequency identification (RFID) transponders (Planet ID GmbH, Essen, Germany), (Zeldovich, 2016) were injected subcutaneously in the dorso-cervical region under isoflurane inhalation anesthesia 1 week before the behavioral testing. At the age of 8 months, C57BL/6J mice were randomly assigned to two experimental groups, the inclusive choice (*N* = 15, *M* = 7, *F* = 8) and the exclusive choice (*N* = 14, *M* = 6, *F* = 8), and introduced to the IntelliCage apparatus. While the IntelliCage 1 accommodated 5 male mice (inclusive choice = 3, exclusive choice = 2), the IntelliCage 2 accommodated 8 males (inclusive choice = 4, exclusive choice = 4). The IntelliCages 3 and 4 accommodated 8 female mice each (inclusive choice = 4, exclusive choice = 4). In line with the 3Rs principles, we adopted recommendations to prevent aggression between the group-housed male mice, aiming to avoid fighting episodes and improve animal welfare. To facilitate species-specific behaviors reducing the prevalence of aggression (Van Loo et al., 2001), we provided environmental enrichment by increasing cage complexity. In particular, we provided transparent tubes (diameter: 4 cm; length: 15 cm) connecting each IntelliCage with a freely accessible extension cage (Figure 1A) (425 × 266 × 155 mm). As spot cleaning when needed, rather than a weekly full cage change, is associated with a lower prevalence of aggression (Lidster et al., 2019), we cleaned either the IntelliCage or the extension cage per time every 10 days, also retaining some clean and dry nesting material and transferring them during cage changes. Additionally, we consistently monitored the animals by behavioral observations (fighting, chasing, mounting, and submissive behavior) and physical evidence (tail wounds, rump and back wounds/hair loss, and urogenital wounds). Adopting the above-mentioned recommendations lets us avoid fighting episodes that could have interfered with the acquisition of behavioral data.

### Behavioral procedures

#### The IntelliCage system

Behavioral testing was conducted in the IntelliCage system (TSE Systems, Bad Homburg, Germany), which is a fully automated cage system designed for the assessment of cognitive abilities in group-housed small rodents (Lipp, 2005; Kiryk et al., 2020; Lipp et al., 2024). The apparatus (Figure 1A) consists of a polycarbonate cage (20.5 cm high, 58 × 40 cm top, 55 × 37.5 cm bottom, Techniplast, 2000P, Buguggiate, Italy) equipped with four triangular operant test chambers (15 × 15 × 21 cm) fitted into each corner. Each chamber contains two drinking bottles, accessible via two round openings that can be opened and closed with motorized doors. Mice that access a chamber are identified by a circular RFID antenna at its entrance, and the duration of their visit is determined by both the antenna reading and a temperature sensor that detects the presence of the animal inside the corner. During a visit, the number and duration of individual nosepokes at each door are recorded using infrared (IR)-beam sensors. Licking episodes at each bottle are monitored using lickometers. Additionally, an extension cage was connected to each IntelliCage via a tube, and behavioral experiments started simultaneously for all animals by opening the connecting tubes. The system has individual controllers, and they are connected to a central PC running the software that permits the design and control of experiments remotely and the analysis of the recorded data (IntelliCage Plus, TSE Systems, Bad Homburg, Germany).

#### Design of the novel appetitively motivated protocols in the IntelliCage

To promote appetitive learning by exploiting the strong preference of C57BL/6J mice for saccharin over plain water (Bachmanov et al., 2001), we developed novel protocols based on the possibility of choosing between saccharin and plain water (Figure 1B). For each corner, one side provided a bottle of plain water (joker side), while the other side had a bottle of sweetened water containing 0.5% saccharin (task side). The joker door opened automatically for 3 seconds at the beginning of any visit during every protocol, while the task door opened for 3 seconds only in response to a nosepoke in a correct corner. Thus, while water was available for free at the joker sides, the mice had to acquire and follow the rules of the respective learning task to obtain sweet rewards at the task sides. To avoid spontaneous bias to respond at task or joker side, the sweetened water bottles were placed on the left side in two corners and on the right side in the two other corners. Mice were assigned to two experimental groups: the *exclusive choice group* (*M* = 6, *F* = 8) and the *inclusive choice control group* (*M* = 7, *F* = 8). A first poke at the joker side prevented the opening of the task door during the same visit in the *exclusive choice group*. Similarly, a first poke at the task side immediately triggered the closing of the joker door, thereby shortening the availability of water. Instead, the task and joker doors operate independently, allowing successful responses on both sides during the same visit in the *inclusive choice control group*. The *exclusive choice* protocol was designed to test whether not having the possibility to choose both saccharin and water as a backup would increase the motivation of the mice to learn to access saccharin.

#### Adaptation phases

Free adaptation: (FA 10, 8 days): Animals were first habituated for 10 days in the IntelliCage environment in a free adaptation stage with all doors open and free access to plain water at any time. During the following 8 days, doors remained constantly open, and each corner provided both a bottle of plain water and a bottle containing 0.5% saccharin solution. This let the mice learn where the water and saccharin were available.

Nosepoke adaptation (NPA, 8 days): All doors were closed by default. The doors hiding plain water opened at the beginning of any visit for 3 seconds. The doors hiding the 0.5% saccharin solution could be opened with a nosepoke once per visit, with time to drink limited to 3 seconds.

#### Learning tasks

Corner preference acquisition (PPRA1, 7 days; PPRA2, 9 days) and reversal learning (PPRR, 6 days): for acquisition training, each mouse was assigned to one correct corner based on its corner preference during NPA (either the second- or third-favorite corner was assigned with a balanced distribution). All doors were closed by default. The doors hiding plain water opened at the beginning of every visit in every corner, while the doors hiding saccharin opened for 3 seconds once per visit only in response to a nosepoke in the correct corner. After cleaning the cages, acquisition training was continued without changing the correct corners (PPRA2). The correct corner was moved to the opposite corner for each mouse in the reversal phase, with conditions for the joker sides remaining the same.

Place time acquisition (PPTA, 11 days) and reversal learning (PPTR, 7 days): as for corner preference acquisition, each mouse is assigned to an initial correct corner with the other corners being incorrect. But the correct corner changed position every 12 hours, moving to the right at 14:00 every day and back to the original position at 02:00. Correct and incorrect corners operated in the same way as during the corner preference task. In the reversal phase, mice had access to the saccharin solution in the corners diagonally opposite the ones assigned in the acquisition stage.

Place time serial reversal (PPTS, 28 days): The protocol consisted of seven alternations between place time acquisition and reversal, each lasting 4 days, starting and ending with an acquisition.

#### Experimental parameters

Post-processing steps were applied to obtain composite variables from the IntelliCage system’s output file (Ma et al., 2023). They include task responses defined as visits to a corner with at least one nosepoke on the task door, and joker responses defined as visits with at least one nosepoke on the joker door and hits stratified into joker and task hits. In this context, we calculated the task response ratio *R*, as indicated below.


$$R=\frac{2\;+\;2^{*}\mathrm{Task}\;\mathrm{responses}}{2\;+\;\mathrm{Joker}\;\mathrm{responses}\;+\;\mathrm{Task}\;\mathrm{responses}\;}$$

This value tends to 0 after many responses exclusively on the joker side and to 2 after a large number of responses exclusively on the task side. A value of 1 indicates the absence of a door preference. We calculated the false rate, which is defined as the percentage of task responses in incorrect corners as a measure of learning and task performance. In the absence of a learning effect, this value is expected to be around 75%, with a significant reduction indicating successful learning of the task rule.

### Statistical analysis

Behavioral data were extracted with the IntelliCage Analyzer software and further processed using Excel. The statistical analysis was conducted using a linear model with sex (male and female) and choice group (inclusive and exclusive) as between-subject factors. Within-subject factors were added as needed to explore the dependence of behavior on time or corner side. Significant interactions were explored by splitting the model. Significant effects of time were further explored using partial models. Variables with strongly skewed distributions or strong correlations between variances and group means were subjected to Box–Cox transformation before statistical analysis, as indicated in figure legends. The significance threshold was set at 0.05. The false discovery rate (FDR) control procedure of Hochberg was applied to groups of conceptually related variables within single tests to correct significance thresholds for multiple comparisons. Similarly, FDR correction was applied during post-hoc testing. One-sample *t*-tests were used to compare values against chance levels. The statistical analyses and graphs were obtained using R version 4.3.0, complemented with the package ggplot2. In line graphs, untransformed data are plotted as mean + SEM with individual data points in the background.

## Results

### Male mice showed a stronger preference for responding exclusively at the saccharin sides during the free adaptation stage

**Figure 2:**
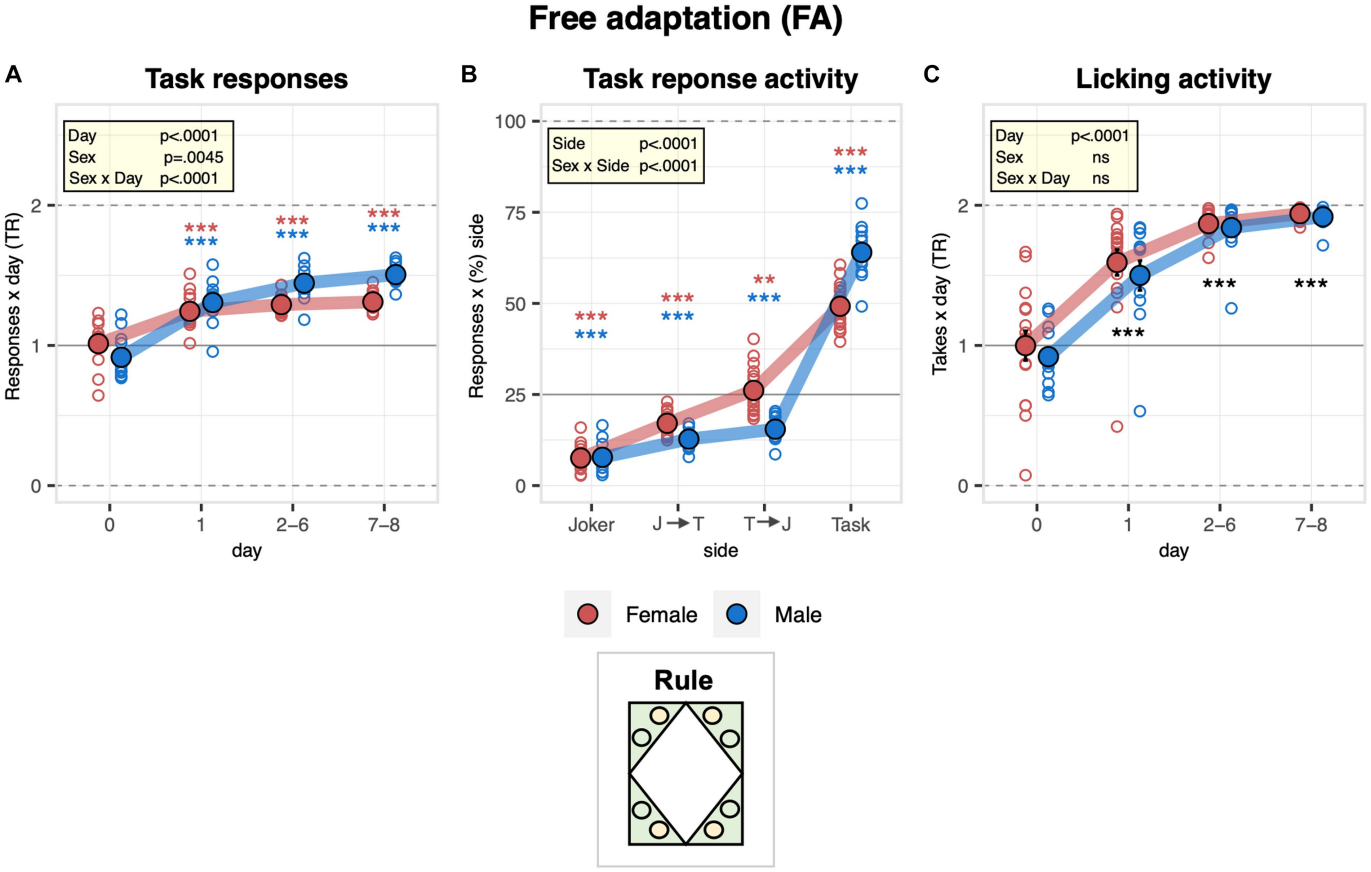
Activity of male and female mice during the free adaptation stage in the IntelliCage. During the free adaptation stage (8 days), saccharin was introduced to task sides for the first time while doors always remained open. During day 0, that indicates the last day of the previous stage (pre-task baseline, all bottles still contained plain water). One-sample *t*-tests against chance (solid horizontal line): ^***^p < 0.001, ^**^p < 0.01, and ^*^p < 0.05 referring to the comparison of pooled groups against chance. One-sample *t*-test results are shown for groups separately in red and blue when a significant interaction with sex is present. **(A)** Response task ratio plotted as a function of day. Ratio defined as (2 + 2 × Task)/(2 + Joker + Task), chance = 1, range = 0–2. Responses defined as visits with poke(s) at task and joker sides, respectively (ANOVA: day *F*_3,72_ = 103.7 *p* < 0.0001 *ω*^2^ = 0.70, sex × day *F*_3,72_ = 12.28 *p* < 0.0001 *ω*^2^ = 0.21, Box–Cox *λ* 1.50). Response task ratio increased more strongly in male mice and stabilized at a level of about 1.4. **(B)** Percentage of responses plotted as a function of side sequence during the visit (joker side only, joker side followed by task side, task side followed by joker side, task side only) and averaged across the entire stage. All except joker side only is counted as a task response, all but task side only is considered a joker response (ANOVA: taste *F*_3,72_ = 288.6 *p* < 0.0001 *ω*^2^ = 0.92, sex × taste *F*_3,72_ = 14.23 *p* < 0.0001 *ω*^2^ = 0.33, Box–Cox *λ* 0.500). Overall, mice preferred to respond exclusively for saccharin while visits with a response plain water were below the chance level. In addition, males more strongly avoided responding to both sides than females and showed a stronger preference for responding exclusively to the task side. **(C)** Drinking task ratio plotted as a function of the day (ANOVA: day *F*_3,72_ = 192.2 *p* < 0.0001 *ω*^2^ = 0.79, Box–Cox *λ* 5.00). The ratio increased rapidly upon the introduction of saccharin to reach levels close to 2, indicating that the mice overall almost exclusively consumed saccharin.

With the free adaptation stage, we aimed to let the mice explore the new environment, learning where the water and saccharin were available. During the pre-task baseline, when all bottles contained water, there was no spontaneous bias to respond at task or joker sides (responses = visits with at least one nosepoke). Overall, mice switched to preferential responding at task sides instantly upon introducing saccharin, males more strongly than females (Figure 2A). They overall preferred to respond exclusively at the saccharin side, while visits with a response to the water side were below the chance level (Figure 2B). In this context, male mice more strongly avoided responding at both sides than female mice and showed a stronger preference for responding exclusively at the task side (Figure 2B). In line with these observations, the drinking preference overall changed rapidly upon the introduction of saccharin. The mice eventually almost exclusively consumed saccharin without evidence of a sex effect (Figure 2C). In accordance, the lick frequency increased strongly and instantly in response to the introduction of saccharin at the task sides without evidence of a sex effect (Supplementary Figure S1).

### The exclusive choice group responded more exclusively for saccharin during the nosepoke adaptation stage, confirming the functioning of the learning protocols

**Figure 3:**
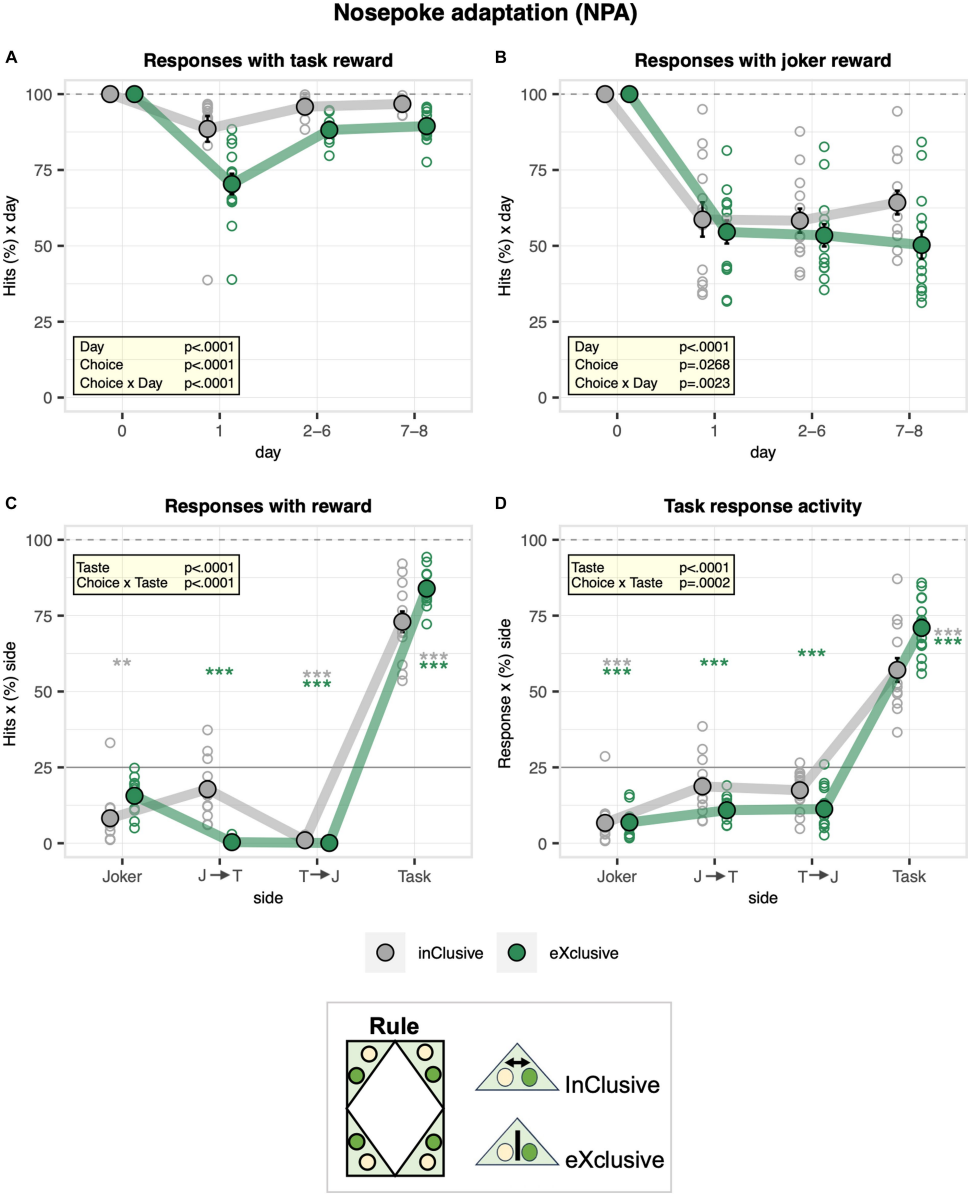
Activity of the inclusive and exclusive experimental groups during the nosepoke adaptation stage. One-sample *t*-tests are shown for groups separately in gray and green when a significant interaction with choice is present: ^***^p < 0.001, ^**^p < 0.01, and ^*^p < 0.05. **(A)** Percentage of nosepokes overlapping with accessibility of saccharin bottles plotted as a function of the day (ANOVA: day *F*_3,69_ = 143.9 *p* < 0.0001 *ω*^2^ = 0.77, choice × day *F*_3,69_ = 17.46 *p* < 0.0001 *ω*^2^ = 0.28, Box–Cox *λ* 4.00). Overall, the percentage of responses with nosepokes overlapping with the accessibility of saccharin bottles dropped when door operation was activated. It dropped more strongly in the exclusive experimental group. **(B)** Percentage of responses with nosepokes overlapping with the accessibility of water bottles plotted as a function of the day (ANOVA: day *F*_3,69_ = 57.08 *p* < 0.0001 *ω*^2^ = 0.58, choice × day *F*_3,69_ = 5.320 *p* = 0.0023 *ω*^2^ = 0.10, Box–Cox *λ* 0.500). Overall, the percentage of nosepokes overlapping with the accessibility of water bottles dropped to 57% when the door operation was activated, without evidence of recovery. The inclusive, experimental group learned to switch faster to the joker side after a first response to the task side. **(C)** Percentage of responses with nosepokes overlapping with accessibility of water and saccharin reward plotted as function of task sequence during the visit (joker side only, joker side followed by task side, task side followed by joker side, and task side only) and averaged across the entire stage (ANOVA: choice × taste *F*_3,69_ = 48.30 *p* < 0.0001 *ω*^2^ = 0.64, Box–Cox *λ* 0.500). Exclusive nosepokes overlapping with accessibility of both water and saccharin were more frequent in the exclusive choice group with dual hits almost never occurring. **(D)** Percentage of responses plotted as function of side sequence during the visit (joker side only, joker side followed by task side, task side followed by joker side, task side only) and averaged across the entire stage (ANOVA: choice × taste *F*_3,69_ = 7.418 *p* = 0.0002 *ω*^2^ = 0.20, Box–Cox *λ* 0.500). The exclusive choice group was more exclusive in its choice to respond for saccharin as intended with the designed protocols.

Following the free adaptation stage, door operation was activated at the task and joker sides during the nosepoke adaptation stage. Overall, the percentage of responses with nosepokes overlapping with the accessibility of saccharin bottles, defined as task hits, dropped when door operation was activated and recovered rapidly to about 93% as mice adapted to the movement of doors (Figure 3A). Moreover, they dropped more strongly in the exclusive group and remained lower throughout the stage, reflecting unsuccessful attempts to drink saccharin after a first response at the joker side (Figure 3A). On the other hand, the percentage of responses with nosepokes overlapping with the accessibility of water bottles, defined as joker hits, overall dropped to 57% when the door operation was activated without evidence of recovery (Figure 3B). This happened since mice could not control the door with nosepokes and came too late when they poked first at the saccharin door and then at the water door. While the exclusive choice group and the control inclusive choice group showed a similar initial drop in the joker hit rate, the control group learned to switch faster to the joker side after a first response to the task side, as indicated by the diverging curves (Figure 3B). Confirming how the designed protocols worked as intended, exclusive hits (either task or joker) were more frequent in the exclusive choice group, with dual hits almost never occurring (Figure 3C). In line with this observation, the exclusive choice group more strongly avoided responding to both sides than the inclusive choice control group, showing a stronger preference for responding exclusively to the task side. Therefore, the exclusive choice group was more exclusive in its choice to respond to saccharin as intended with the designed protocols (Figure 3D). During the nosepoke adaptation stage, we also observed some sex effects, which were in line with our observations made during the free adaptation stage. While the hit rate of males dropped less strongly at task sides (Supplementary Figure S2A) when the new protocol was introduced, it showed a stronger transient drop than in females at joker sides (Supplementary Figure S2B). Sexes were similar with respect to the overall distribution of hit rates across sides during nosepoke adaptation (Supplementary Figure S2C), but as during free adaptation, males continued to respond more exclusively than females at task sides (Supplementary Figure S2D).

### The presence of saccharin motivates mice to learn the corner preference acquisition and reversal tasks

**Figure 4:**
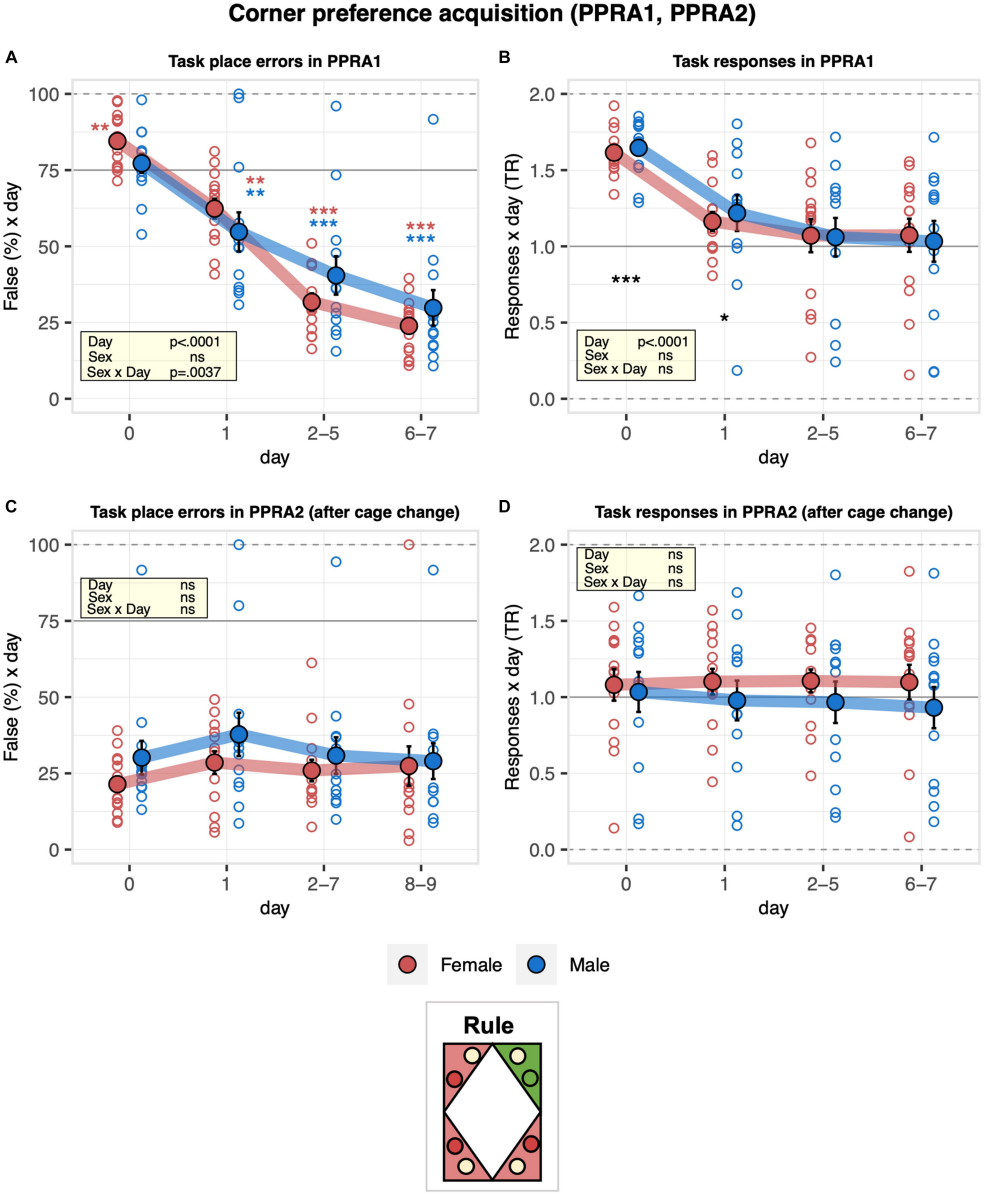
Learning performance of males and females in the corner preference acquisition stage. During corner preference acquisition 1 (PPRA1, 7 days), water was available at joker sides in all corners, but saccharin could only be obtained at the task side of a single target corner, which remained the same throughout the task. Corner preference acquisition 2 (PPRA2, 9 days) continued with the same target corner as in corner preference acquisition 1 after cleaning the cages. Percentage of place errors corresponds to task responses to incorrect corners plotted as a function of the day, with Day 0 corresponding to the last 2 days of nosepoke adaptation with saccharin still available in all corners. Response task ratio was plotted as a function of the day. Ratio defined as (2 + 2 × Task)/(2 + Joker + Task), chance = 1, range = 0–2. One-sample *t*-tests against chance (solid horizontal line): ^***^p < 0.001, ^**^p < 0.01, and ^*^p < 0.05 referring to the comparison of pooled groups against chance. One-sample *t*-test results are shown for groups separately in red and blue when a significant interaction with sex is present. **(A)** Percentage of place errors during PPRA1 (ANOVA: day *F*_3,69_ = 157.6 *p* < 0.0001 *ω*^2^ = 0.66, sex × day *F*_3,69_ = 4.925 *p* = 0.0037 *ω*^2^ = 0.05). Place errors were slightly above chance during pre-task baseline and decreased robustly below chance indicating the mice successfully learned the place rule. Error numbers decreased somewhat more slowly in males than females. **(B)** Response task ratio during PPRA1 (ANOVA: day *F*_3,69_ = 68.12 *p* < 0.0001 *ω*^2^ = 0.41, Box–Cox *λ* 3.00). Response task ratio decreased strongly at the beginning of the learning task and continued to decrease during the task, reaching near indifference at the end of training. **(C)** Percentage of place errors during PPRA2 (ANOVA: day *F*_3,69_ = 1.585 ns, sex *F*_1,23_ = 1.254 ns, Box–Cox *λ* 0.500). There was no statistical evidence for an effect of cage cleaning on place error rate. In addition, there was no evidence of a sex effect on the overall learning performance. **(D)** Response task ratio during PPRA2 (ANOVA: day *F*_3,69_ = 0.1422 ns, sex *F*_1,23_ = 0.4463 ns, Box–Cox *λ* 2.00). Response task ratio remained near indifference without evidence of a change over time and without evidence of a sex effect.

**Figure 5:**
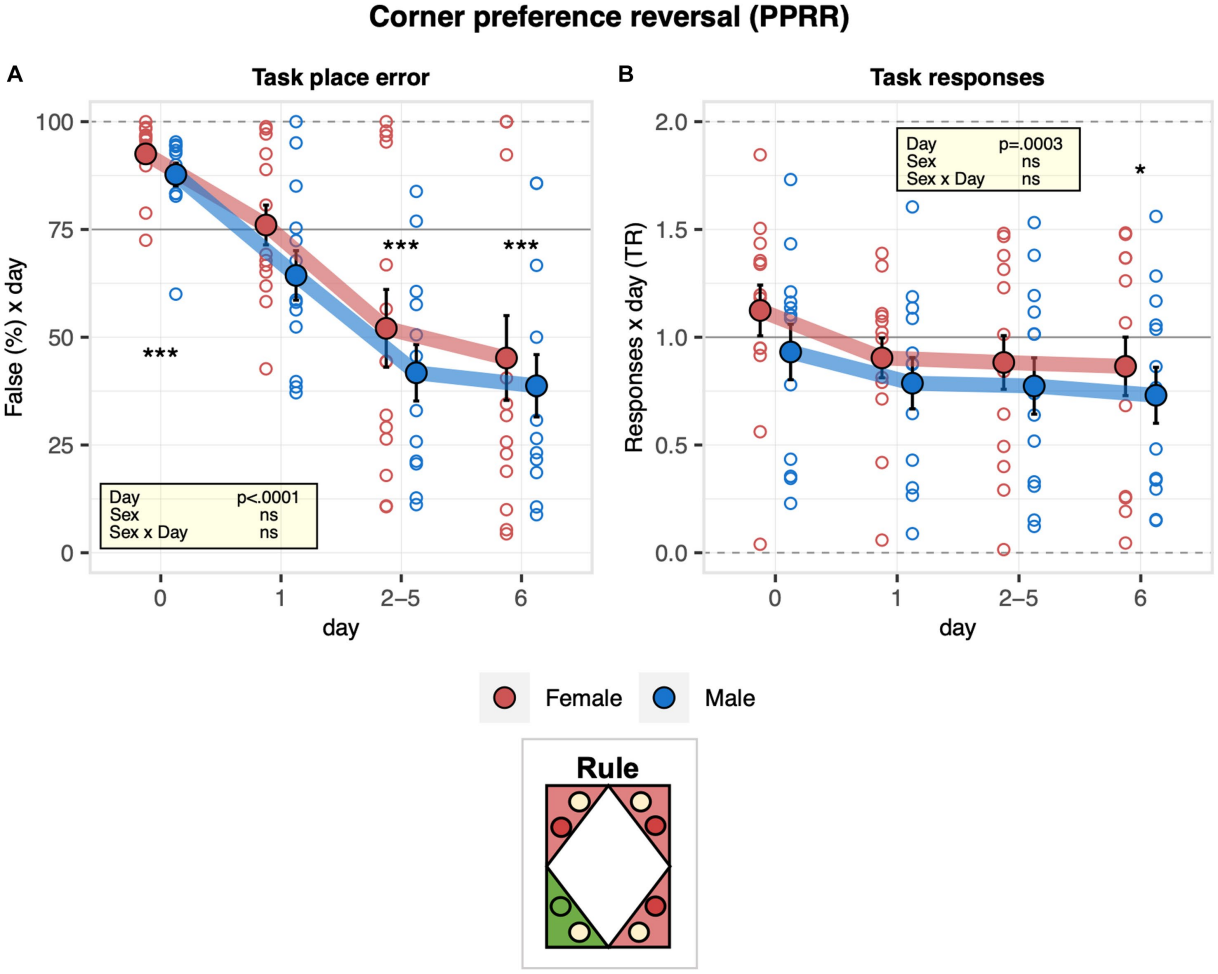
Learning performance of males and females in the corner preference reversal stage. During corner preference reversal (PPRR, 6 days), the target corner was opposite to the one used in corner preference acquisition 1 and 2 (PPRA1, PPRA2). One-sample *t*-tests against chance (solid horizontal line): ^***^p < 0.001, ^**^p < 0.01, and ^*^p < 0.05 referring to the comparison of pooled groups against chance. **(A)** Percentage of place errors corresponding to task responses to incorrect corners plotted as a function of the day with day 0 corresponding to the last 2 days of corner preference acquisition 2 (ANOVA: day *F*_3,69_ = 50.62 *p* < 0.0001 *ω*^2^ = 0.40 sex *F*_1,23_ = 1.151 ns). Place error rates decreased robustly and reached a level below chances, indicating how the mice learned the new rule without evidence for a sex effect on learning performance. **(B)** Response task ratio plotted as a function of the day, with day 0 corresponding to the last 2 days of corner preference acquisition 2. Ratio defined as (2 + 2 × Task)/(2 + Joker + Task), chance = 1, range = 0–2 (ANOVA: day *F*_3,69_ = 7.027 *p* = 0.0003 *ω*^2^ = 0.04). Mice overall started to respond preferentially at the water sides.

The learning performance of C57BL/6J male and female mice was first addressed in the corner preference acquisition and reversal tasks. During corner preference acquisition 1 (PPRA1), place errors were slightly above chance during the pre-task baseline when all corners were still rewarded with saccharin and decreased robustly below chance, indicating the mice successfully learned the place rule (Figure 4A). While there was no evidence for an overall sex effect on performance, error numbers decreased somewhat more slowly in males than females (Figure 4A). The response task ratio decreased strongly at the beginning of the learning task and continued to decrease during the task, reaching near indifference at the end of training (Figure 4B). Corner preference acquisition 2 (PPRA2) continued with the same target corner as corner preference acquisition 1 (PPRA1) after cage cleaning. To note, there was no statistical evidence for an effect of cage change on place error rate, indicating that cage cleaning was not interfering with their performance (Figure 4C). In addition, there was no evidence of a sex effect on the overall learning performance (Figure 4C). In line with this observation, the response task ratio remained near indifference without evidence of a change over time. In addition, no evidence of a sex effect on the response task ratio was observed (Figure 4D). Looking at the reversal stage, place error rates decreased robustly and reached levels below chance, indicating that the mice learned the new rule. There was no evidence for a sex effect on learning performance (Figure 5A). To note, the mice overall shifted toward responding preferentially at the water sides, as suggested by the decreased response task ratio in the corner preference reversal stage (Figure 5B).

### Mice learn the place time task, with males performing more poorly and preferentially responding at water sides compared to females

**Figure 6:**
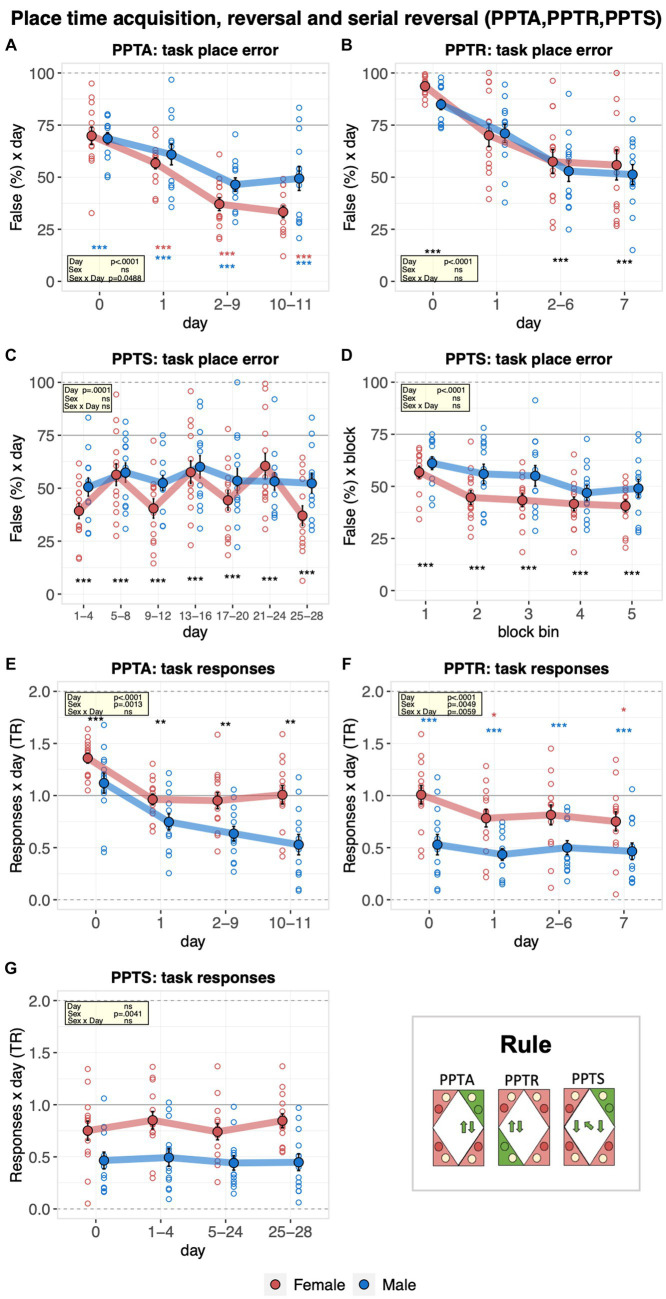
Learning performance of males and females in the place time acquisition, reversal, and serial reversal stages. During the place time acquisition (PPTA, 11 days), the target corner moved to the right at 14:00 every day and back to the original position at 02:00. During the place time reversal (PPTR, 7 days), a new corner pair, with target corner opposite to the one used in time place acquisition and again moved to the right at 14:00 every day and back to the original position at 02:00, was defined. During the place time serial reversal (PPTS, 28 days), 7 alternations between place time acquisition and reversal, each lasting for 4 days, starting and ending with the acquisition, were defined. One-sample *t*-tests against chance (solid horizontal line): ^***^p < 0.001, ^**^p < 0.01, and ^*^p < 0.05 referring to the comparison of pooled groups against chance. One-sample *t*-test results are shown for groups separately in red and blue when a significant interaction with sex is present. **(A)** Percentage of place errors during PPTA corresponding to task responses to incorrect corners plotted as a function of the day with day 0 corresponding to the last 2 days of nosepoke adaptation III with saccharin available in all corners (ANOVA: day *F*_3,69_ = 38.24 *p* < 0.0001 *ω*^2^ = 0.44, sex × day *F*_3,69_ = 2.758 *p* = 0.0488 *ω*^2^ = 0.04). Overall, place errors decreased robustly indicating how the mice learned the place time acquisition rule. Males made significantly more place errors from the second day onward. **(B)** Percentage of place errors during PPTR corresponding to task responses to incorrect corners plotted as a function of the day with day 0 corresponding to the last 2 days of place time acquisition (ANOVA: day *F*_3,69_ = 37.33 *p* < 0.0001 *ω*^2^ = 0.42). Overall, mice learned the place acquisition rule. **(C)** Percentage of place errors during PPTS corresponding to task responses to incorrect corners plotted as a function of the day (7 × 4 days, average of each alternation, ANOVA, sex × day *F*_6,138_ = 1.930 *p* = 0.0801 *ω*^2^ = 0.02). There was no general learning of the place time serial reversal task. **(D)** Percentage of place errors during PPTS corresponding to task responses to incorrect corners plotted as a function of block bin (first, second, third, fourth, and fifth 20% responses in each alternation, averaged across alternations, ANOVA: block bin *F*_1,104_ = 55.03 *p* < 0.0001 *ω*^2^ = 0.12). A robust decrease of place error rate within each task was observed. **(E)** Response task ratio during PPTA plotted as a function of the day, with day 0 corresponding to the last 2 days of nosepoke adaptation III with saccharin available in all corners. Ratio defined as (2 + 2 × Task)/(2 + Joker + Task), chance = 1, range = 0–2 (ANOVA: sex *F*_1,23_ = 13.40 *p* = 0.0013 *ω*^2^ = 0.33). Response task ratio of male mice dropped at task onset, continued to decrease during the task and reached levels clearly indicating preferential responding at water sides. **(F)** Response task ratio during PPTR plotted as a function of the day with day 0 corresponding to the last 2 days of place time acquisition (ANOVA: sex *F*_1,23_ = 9.705 *p* = 0.0049 *ω*^2^ = 0.26; sex × time bin *F*_3,69_ = 4.531 *p* = 0.0059 *ω*^2^ = 0.01). Response task ratio of male mice remained at very low levels throughout the task, indicating persistent preferential responding at water sides compared to female mice. **(G)** Response task ratio plotted as a function of the day (7 × 4 days, average of each alternation). (ANOVA: sex *F*_1,23_ = 10.14 *p* = 0.0041 *ω*^2^ = 0.27). Response task ratio of male mice remained consistently below that of females.

The learning performance of C57BL/6J male and female mice was then evaluated in the place time acquisition and reversal tasks. Overall, place errors decreased robustly, indicating that the mice learned the place time acquisition rule, reaching a plateau on the second experimental day (Figure 6A). From the second day onward, males made more place errors (Figure 6A). Looking at the place time reversal stage, mice also learned the new place rule, as indicated by the robust decrease in the place error rates during the task (Figure 6B). In contrast, there was no improvement across repeated goal changes in the place time serial reversal task, indicating that the mice could not learn to adapt more efficiently to the changing pairs of target corners (Figure 6C). However, there was a robust decrease in place error rate within each task as mice adapted to the new corner pair (Figure 6D). During the place time task, choices to respond at water or saccharin doors showed a striking sex difference. Male mice switched to preferential responding for water at task onset, and their responding at task sides further decreased as the task progressed. By contrast, females still responded preferentially for saccharin at baseline and did not develop a preference for responding at water sides throughout the task (Figure 6E). Throughout the reversal stage, males preferred to respond to water. Females also shifted toward preferential responses for water, but clearly fewer than males (Figure 6F). In line with these observations, the preference of male mice to respond to water remained clearly stronger than that of females during the place time serial reversal stage (Figure 6G).

### Preference to respond for saccharin is generally lost with the introduction of the first task

**Figure 7:**
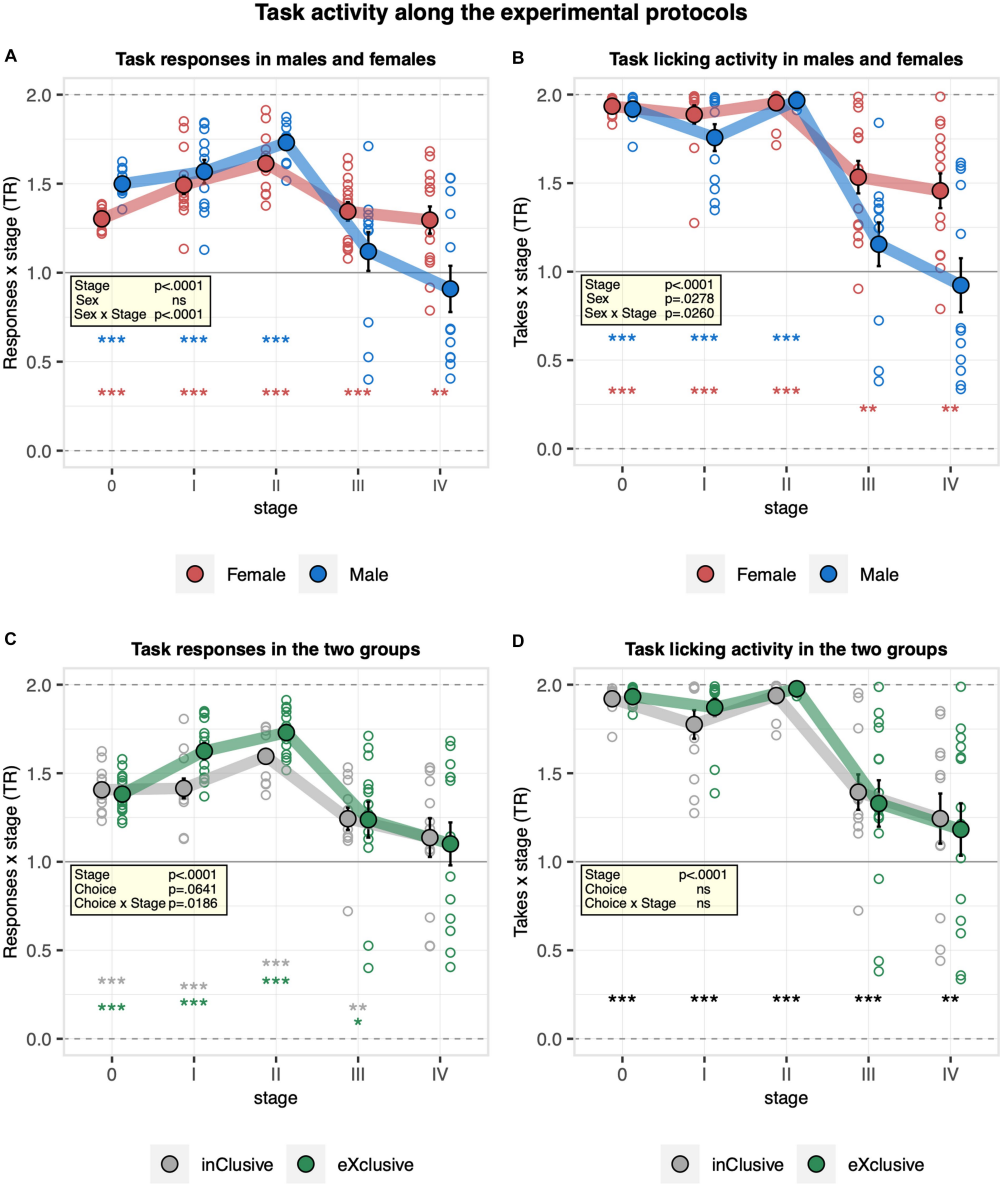
Activity of the mice at the task sides during nosepoke adaptation stages before, between, and after learning tasks. Response task ratio was plotted as a function of stage, each corresponding to the last 2 days of a phase (0 free adaptation saccharin/water, I first nosepoke adaptation, II continued pre-learning nosepoke adaptation, III nosepoke adaptation after corner preference training, and IV final nosepoke adaptation after time place training). Ratio defined as (2 + 2 × Task)/(2 + Joker + Task), chance = 1, range = 0–2. The licking task ratio was calculated based on takes = responses with drinking at task and joker sides, respectively. One-sample *t*-tests against chance (solid horizontal line): ^***^p < 0.001, ^**^p < 0.01, and ^*^p < 0.05 referring to the comparison of pooled groups against chance. One-sample *t*-test results are shown for groups separately when a significant interaction with either sex or choice is present. **(A)** Response task ratio in males and females (ANOVA: phase *F*_4,88_ = 33.38 *p* < 0.0001 *ω*^2^ = 0.47, sex × phase *F*_4,88_ = 7.275 *p* < 0.0001 *ω*^2^ = 0.15, Box–Cox *λ* 3.50). The response task ratio increased throughout the pre-learning stages but dropped after the learning stages, without evidence for recovery during nosepoke adaptation interludes. It was slightly higher in males during the pre-learning stages but dropped more strongly after learning than in females. **(B)** Licking task ratio in males and females (ANOVA: sex × phase *F*_4,88_ = 2.909 *p* = 0.0260 *ω*^2^ = 0.05, Box–Cox *λ* 5.00). Males showed a stronger decrease in the drinking task ratio after the learning stages. **(C)** Response task ratio in the inclusive and exclusive groups (ANOVA: choice × phase *F*_4,88_ = 3.130 *p* = 0.0186 *ω*^2^ = 0.06, Box–Cox *λ* 3.50). The response task ratio was higher in the exclusive choice group during the pre-learning stages, but the effect was lost after the learning stages. **(D)** Drinking task ratio in the inclusive and exclusive groups (choice *F*_1,22_ = 0.2115 ns, Box–Cox *λ* 5.00). No evidence for a choice group effect on the drinking task ratio was observed.

To examine the behavior of mice across the different experimental protocols, we analyzed their overall task activity throughout nosepoke adaptation stages before, between, and after learning tasks. At the transition from free to nosepoke adaptation, lick numbers dropped by about 50% and remained stable thereafter, with only very small decreases after learning stages without evidence for a consistent effect of sex on lick numbers (Supplementary Figure S3A). Moreover, no evidence for a choice group effect on the overall lick number was observed (Supplementary Figure S3B). Looking at the response numbers, they overall increased strongly at the transition from free to nosepoke adaptation to decrease again after the learning stages without statistical evidence for a sex effect (Supplementary Figure S3C). They were also similar in the two experimental groups (Supplementary Figure S3D). Looking specifically at the preference to respond to saccharin, it increased throughout the pre-learning stages but dropped after the learning stages without evidence for recovery during nosepoke adaptation interludes (Figure 7A). Moreover, it was slightly higher in males during the pre-learning stages but dropped more strongly after learning than in females (Figure 7A). In line with this observation, a stronger decrease in the preference for drinking saccharin after the learning stages was observed in males compared to females (Figure 7B). These data indicate how the motivation of males to respond to saccharin did not fully recover when saccharin became available in all corners again after spatial learning tasks. Looking at the two experimental groups, the preference to respond to saccharin was slightly higher in the exclusive choice group during pre-learning nosepoke adaptation, but the effect was lost after the learning stages (Figure 7C). No evidence for a choice group effect on the preference for drinking saccharin was detected (Figure 7D).

### No evidence for better learning performance in the exclusive choice group

The learning performance of the two experimental groups was addressed in the corner preference acquisition/reversal tasks and place time acquisition/reversal/serial reversal tasks. There was no statistical evidence for improved performance of the exclusive choice group during corner preference acquisition 1 (Supplementary Figure S4A) or corner preference acquisition 2 (Supplementary Figure S4B). In line with this observation, no statistical evidence for an enhancing effect of the exclusive choice protocol on the preference to respond for saccharin was observed in corner preference 1 (Supplementary Figure S4C) or corner preference 2 (Supplementary Figure S4D) acquisition. During the reversal stage, the two experimental groups were similar in terms of both learning performance (*F*_1,23_ = 0.0632 ns, Supplementary Figure S4E) and preference to respond to saccharin (Supplementary Figure S4F). Moreover, there was no evidence for improved performance or learning rate in the exclusive choice group during the place time acquisition (Supplementary Figure S5A), reversal (Supplementary Figure S5B), and serial reversal (Supplementary Figures S5C, D) tasks. In line with this observation, the two experimental groups showed a similar preference to respond to saccharin in the acquisition (Supplementary Figure S5E), reversal (Supplementary Figure S5F), and serial reversal (Supplementary Figure S5G) stages.

## Discussion

In the present study, we compared task engagement and learning performance of male and female C57BL/6J mice in the IntelliCage in a set of increasingly difficult appetitively motivated spatial learning tasks. In all tasks, successful learning gave access to a sweet reward, while plain water was freely available to prevent water deprivation in poor learners and to create a purely appetitive incentive for learning. In line with a previous study (Bramati et al., 2023), our results confirm that this purely appetitive incentive is sufficient to drive learning in simple but not in more demanding IntelliCage tasks. In addition, we observed that male mice, despite being attracted more strongly by the sweet reward when it was available for free, were less successful than females in engaging in learning to obtain access to sweet reward and performed more poorly in demanding IntelliCage tasks. Finally, we found that a modification of the protocol enforcing an exclusive choice of either plain water or sweet water reward failed to improve performance in female and male mice, even though it prevented the use of plain water as backup during incorrect responses.

Given the well-documented sex differences in both physiology and behavior, it is mandatory that both female and male subjects are tested to capture sex-dependent aspects of disease mechanisms and when mouse models are used for modeling a human population (Shansky, 2018). Thus, to be valid, cognitive tests must be applicable to subjects of both sexes. Given that the IntelliCage system is generally suitable for testing mice of both sexes (Kiryk et al., 2020; Lipp et al., 2024), we deemed it necessary to assess task performance in our appetitively motivated protocols for IntelliCage not only in female but also in male mice. The attractiveness of saccharin, the sweet reward used in our study, to male C57BL/6 mice is well-documented (Bachmanov et al., 2001). As expected, there was no evidence of a sex difference in the almost exclusive choice to drink saccharin solution when both saccharin solution and plain water were freely available during the adaptation stages of our experiment. Because saccharin consumption depends not only on motivation but also on learning success, we used response preference, which also includes nosepokes without licking, as a measure of the motivation to engage in learning tasks. The baseline preference of males for responding at saccharin sides during baseline conditions was even slightly stronger than in female mice. This confirmed that saccharin as a sweet reward was sufficiently attractive to mice of both sexes.

However, when we evaluated learning performance in the set of learning tasks, we observed that the performance of males deteriorated even more rapidly with increasing task difficulty than that of female mice. As already detailed in the introduction section, there is little evidence for a genuine disadvantage of male mice relative to females in learning spatial tasks, and it has been shown previously that male mice of various strains learn challenging spatial tasks well in IntelliCage if they are motivated by water deprivation (Endo et al., 2011). But evidence is emerging that sex differences play a role in value-based decision-making (van den Bos et al., 2013b; Orsini and Setlow, 2017; Shansky, 2018; Grissom and Reyes, 2019; Chen et al., 2021; Cox et al., 2023). This is relevant because the spatial IntelliCage protocols evaluated in the present study rely fully on appetitive motivation, thereby eliminating the need to secure sufficient liquid intake in the interest of body homeostasis as a powerful driver of learning. As a consequence of this design and unlike in conventional IntelliCage tasks, value-based decision-making becomes the main driver of learning and adapting behavior to the changing location of reward. Particularly relevant to our specific setting are observations that motivation to engage in a task is modulated by action value more strongly in female than in male mice (Cox et al., 2023) and that male mice can be more prone than females to adhere to exploratory choice patterns in value-based decision-making tasks (Chen et al., 2021). Engaging in an exploratory response pattern across corners in our appetitively motivated spatial IntelliCage tasks reduces the success rate of responding for saccharin, and this may potentiate the impact of such sex differences on task motivation and learning performance. This interpretation is supported by our observation that task performance and preference to respond to saccharin decreased in parallel. The fact that male mice perform worse in some of the protocols presented here is a limitation that needs to be addressed by improving the protocols. On the other hand, the ability of these protocols to pick up sex differences in decision-making that are not evident in aversively motivated conventional or IntelliCage tasks also indicates that they may also be more suitable to detect alterations of decision-making which may be relevant phenotypic changes in mouse models of brain disease (Perry and Kramer, 2015).

We speculated that having the option to first respond for saccharin and to switch to plain water as a backup after not being rewarded with saccharin in an incorrect corner could lower the cost of incorrect responses and reduce the motivation to learn the task rule. Therefore, we tested a modification of the protocol enforcing an exclusive choice of either plain water or sweet reward during every visit, thereby preventing using plain water as backup during incorrect responses. Our results provide no evidence for a consistent beneficial effect of this modification on task performance. Most likely, this is due to the fact that the mice spontaneously tended to make exclusive responses either at the saccharin or plain water side. Even when allowed and rewarded, double responses for both saccharin solution and plain water were infrequent already during baseline conditions (Figures 3C,D). When challenged by increasingly difficult learning tasks, the mice reduced response for saccharin completely and independently of the protocol and did not adopt a double responding strategy. Obviously, the perceived value of plain water as a backup was too small for the animals to have a significant negative impact on learning motivation.

We deliberately chose saccharin as a sweet reward instead of sucrose because of the metabolic effects that may be induced by the prolonged consumption of a caloric reward on body weight and enzymatic activity (Black and Moyer, 1998). The strain dependence of the preference for saccharin in mice (Bachmanov et al., 2001) is a potential further limitation of the protocols proposed in the present study. In addition, experimental manipulations in mouse models of neurodegenerative disease may alter reward processing at a basic level and thereby compromise the attractiveness of saccharin as a reward (Perry and Kramer, 2015). Therefore, the baseline preference to respond and consume saccharin solution will need to be checked carefully during the adaptation stages in any study using these protocols. Saccharin may need to be replaced by another tastant or by sucrose—or in some cases, one may even need to revert to protocols that use water deprivation as a negative incentive for learning.

In conclusion, IntelliCage protocols which are based on sweet rewards and prevent water deprivation in poor learners by providing continuous access to water, permit to optimize animal welfare and refine the assessment of learning in mouse models following the 3R principles (replace, reduce, refine). However, the validity of such learning tasks still needs to be improved. Learning engagement also needs to be secured in more demanding learning tasks by modifying sweet reward-based protocols in ways that provide a stronger incentive for learning in female mice and even more so in male mice that are less willing to engage in learning for a sweet reward. This could, for example, be achieved by attaching a price tag to the constantly available water to make it less attractive and to create a double incentive for learning. Indeed, we have recently found that introducing a disincentive component either by adding bitter-tasting quinine to the freely available water or by reducing the probability of water delivery at joker sides indeed improves motivation and performance of female mice in challenging spatial tasks in IntelliCage (Ma et al., 2023). However, whether such an approach could also motivate male mice to learn difficult tasks remains to be shown.

## Data availability statement

The raw data supporting the conclusions of this article will be made available by the authors, without undue reservation.

## Ethics statement

All the animal experiments were carried out at the Institute of Anatomy, University of Zürich in accordance with the European legislation (Directive 2010/63/EU) and having been approved by the veterinary office of the Canton of Zürich (License number 060/2021). The study was conducted in accordance with the local legislation and institutional requirements.

## Author contributions

MN: Investigation, Writing – original draft, Writing Review & Editing, Visualization. GB: Investigation, Writing – original draft. ACS: Writing – original draft. DW: Conceptualization, Data curation, Formal analysis, Funding acquisition, Writing – original draft, Writing Review & Editing, Visualization.

## Supplementary material

SUPPLEMENTARY FIGURE S1 Licking frequency of male and female mice during the free adaptation stage in the IntelliCage. Average licks per hour plotted as function of day with Day 0 corresponding to the last day of the previous stage (pre-task baseline, when all bottles still contained plain water) (ANOVA: day F3,72=21.34 *p* <.0001 ω²=.18, sex F1,24=1.236 ns). Lick frequency increased instantly in response to the introduction of saccharin at the task sides. There was no evidence for a sex effect on lick frequency, suggesting that male and female mice consumed similar amounts of liquid.

SUPPLEMENTARY FIGURE S2 Activity of male and female mice during the nosepoke adaptation stage. One-sample t-test results shown for groups separately in red and blue when a significant interaction with sex is present: ****p*<.001 ***p*<.01 **p*<.05. **A)** Percentage of nosepokes overlapping with accessibility of saccharin bottles plotted as function of day (ANOVA: day F3,69=143.9 p<.0001 ω²=.77, sex F1,23=6.040 p=.0219 ω²=.17, sex x day F3,69=2.571 ns, sex x choice F1,23=1.802 ns, Box-Cox λ 4.00). Percentage of nosepokes overlapping with accessibility of saccharin bottles dropped more strongly in females but sexes became indistinguishable by the end of the stage. **B)** Percentage of responses with nosepokes overlapping with accessibility of water bottles plotted as function of day (ANOVA: day F3,69=57.08 p<.0001 ω²=.58, sex F1,23=.3964 ns, sex x day F3,69=3.550 p=.0188 ω²=.06, sex x choice F1,23=1.110 ns, Box-Cox λ .500). Percentage of responses with nosepokes overlapping with accessibility of water bottles dropped more strongly in males but soon recovered to levels slightly higher than females. **C)** Percentage of responses with nosepokes overlapping with accessibility of water and saccharin reward plotted as function of task sequence during the visit (joker side only, joker side followed by task side, task side followed by joker side, task side only) and averaged across the entire stage (ANOVA: sex x taste F3,69=2.546 ns, sex x choice x taste F3,69=2.560 ns, Box-Cox λ .500). **D)** Percentage of responses plotted as function of side sequence during the visit (joker side only, joker side followed by task side, task side followed by joker side, task side only) and averaged across the entire stage (ANOVA: sex x taste F3,69=7.355 p=.0002 ω²=.20, sex x choice x taste F3,69=.9204 ns, Box-Cox λ .500). Males more strongly avoided to respond at both sides than females and showed a stronger preference for responding exclusively at the task side.

SUPPLEMENTARY FIGURE S3 Activity of the mice during nosepoke adaptation stages before, between and after learning tasks. **A, B)** Average licks per day plotted as function of phase each corresponding to the last 2 days of a stage (0 free adaptation saccharin / water, I first nosepoke adaptation, II continued pre-learning nosepoke adaptation, III nosepoke adaptation after corner preference training, IV final nosepoke adaptation after time place training; ANOVA: phase F4,88=46.46 *p*<.0001 ω²=.47, Box-Cox λ .500). At the transition from free to nosepoke adaptation, lick numbers dropped by about 50% and remained stable thereafter with only very small decreases after learning stages, without evidence for a consistent effect of sex on lick numbers. No evidence for a choice group effect on the overall lick number was observed. **C, D)** Average responses per hour plotted as function of phase (ANOVA: phase F4,88=60.69 *p*<.0001 ω²=.50, Box-Cox λ .500). Overall, responses increased strongly at the transition from free to nosepoke adaptation to decrease again after the learning stages without statistical evidence for a sex effect. Responses were also similar in the two experimental groups.

SUPPLEMENTARY FIGURE S4 Learning performance of the inclusive and exclusive groups in the corner preference acquisition and reversal stages. During corner preference acquisition 1 (PPRA1, 7 days), water was available at joker sides in all corners, but saccharin could only be obtained at the task side of a single target corner which remained the same throughout the task. Corner preference acquisition 2 (PPRA2, 9 days) continued with same target corner as in corner preference acquisition 1 after cleaning the cages. During corner preference reversal (PPRR, 6 days) the target corner was defined as opposite to the one used in corner preference acquisition 1 and 2. Ratio defined as (b + 2 x Task) / Proofs_Legends (b + Joker + Task), b=2, chance = 1, range = 0-2. One-sample t-tests against chance (solid horizontal line): ****p*<.001 ***p*<.01 **p*<.05 referring to the comparison of pooled groups against chance. **A)** Percentage of place errors in PPRA1 corresponding to task responses to incorrect corners plotted as function of day with Day 0 corresponding to the last 2 days of joker adaptation with saccharin still available in all corners (ANOVA: choice F1,23=.0326 ns). **B)** Percentage of place errors in PPRA2 corresponding to task responses to incorrect corners plotted as function of day with Day 0 corresponding to the last 2 days of corner preference acquisition 1 (ANOVA: choice F1,23=.1233 ns, Box-Cox λ .500). **C)** Response task ratio plotted as function of day in PPRA1 with Day 0 corresponding to the last 2 days of joker adaptation with saccharin still available in all corners. (ANOVA: choice F1,23=2.277 ns, Box-Cox λ 3.00). **D)** Response task ratio plotted as function of day in PPRA2 with Day 0 corresponding to the last 2 days of corner preference acquisition 1 (ANOVA: choice F1,23=.4011 ns, Box-Cox λ 2.00). **E)** Percentage of place errors during PPRR corresponding to task responses to incorrect corners plotted as function of day with Day 0 corresponding to the last 2 days of corner preference acquisition 2 (ANOVA: choice F1,23=.0632 ns). **F)** Response task ratio plotted as function of day in PPRR with Day 0 corresponding to the last 2 days of corner preference acquisition 2 (ANOVA: choice F1,23=.2247 ns).

SUPPLEMENTARY FIGURE S5 Learning performance of the inclusive and exclusive groups in the place time acquisition, reversal and serial reversal stages. During place time acquisition (PPTA, 11 days), the target corner moved to the right at 14:00 every day and back to the original position at 02:00. During place time reversal (PPTR, 7 days), a new corner pair was defined with target corner opposite to the one used in time place acquisition and again moving to the right at 14:00 every day and back to the original position at 02:00. During place time serial reversal (PPTS, 28 days), 7 alternations between place time acquisition and reversal each lasting for 4 days, starting and ending with acquisition, were defined. One-sample t-tests against chance (solid horizontal line): ****p*<.001 ***p*<.01 **p*<.05 referring to the comparison of pooled groups against chance. **A)** Percentage of place errors during PPTA corresponding to task responses to incorrect corners plotted as function of day with Day 0 corresponding to the last 2 days of joker adaptation III with saccharin available in all corners (ANOVA: choice F1,23=2.463 ns). **B)** Percentage of place errors during PPTR corresponding to task responses to incorrect corners plotted as function of day with Day 0 corresponding to the last 2 days of time place acquisition (ANOVA: choice F1,23=.7869 ns). **C)** Percentage of place errors corresponding to task responses to incorrect corners plotted as function of day (7x4 days, average of each alternation) during PPTS (ANOVA: choice F1,23=2.106 ns). **D)** Percentage of place errors corresponding to task responses to incorrect corners plotted as function of block bin (first, second, third, fourth and fifth 20% responses in each alternation, averaged across alternations) during PPTS (ANOVA: choice F1,23=1.516 ns). **E)** Response task ratio plotted as function of day during PPTA with Day 0 corresponding to the last 2 days of joker adaptation III with saccharin available in all corners. Ratio defined as (2 + 2 x Task) / (2 + Joker + Task), chance = 1, range = 0-2 (ANOVA: choice F1,23=1.626 ns). **F)** Response task ratio plotted as function of day during PPTR with Day 0 corresponding to the last 2 days of time place acquisition. (ANOVA: choice F1,23=1.482 ns). **G**) Response task ratio plotted as function of time bin (7x4 days, average of each alternation) during PPTS. (ANOVA: choice F1,23=1.550 ns).
