# Supplementary figures and images for: Appetitively motivated tasks in the IntelliCage reveal a higher motivational cost of spatial learning in male than female mice
Source: Front Behav Neurosci. 2024 Feb 29;18:1270159. doi: 10.3389/fnbeh.2024.1270159 (PMC10938600; doi:10.3389/fnbeh.2024.1270159)

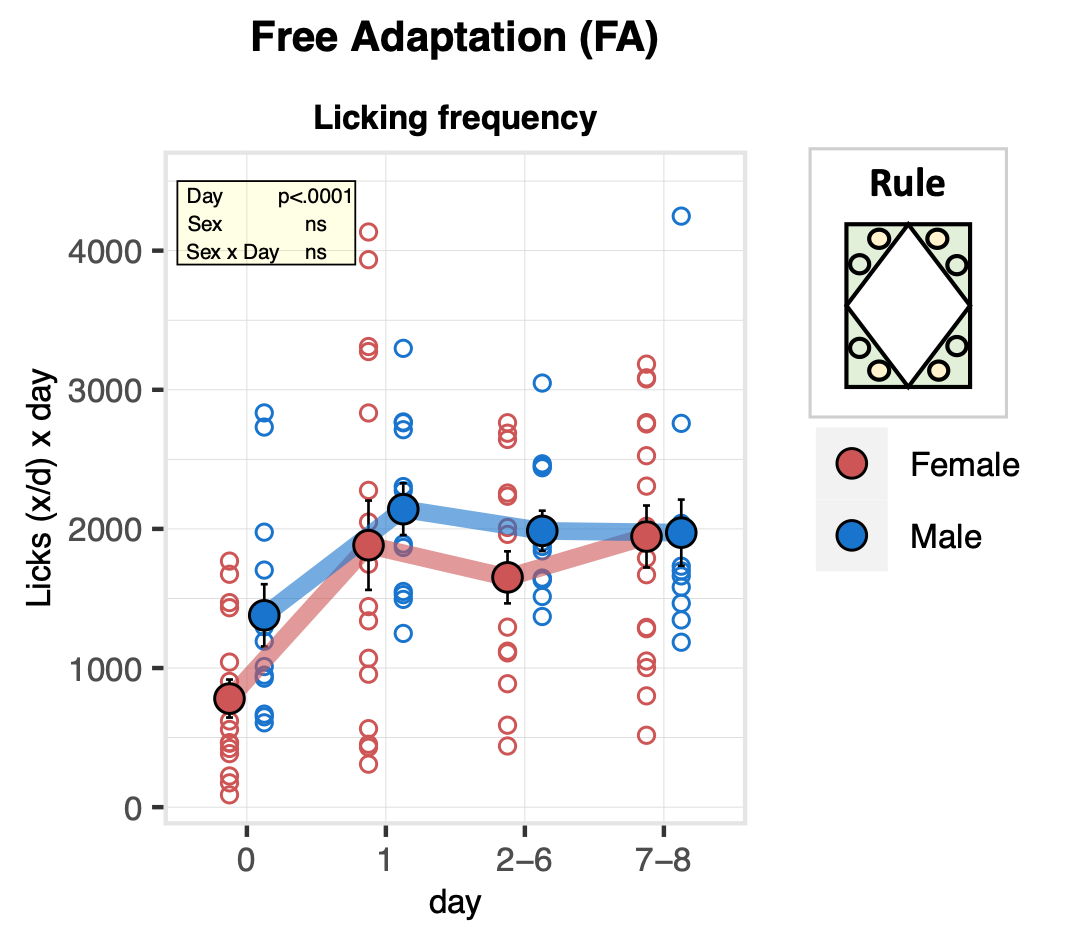

Supplement: SUPPLEMENTARY FIGURE S1 — Licking frequency of male and female mice during the free adaptation stage in the IntelliCage. Average licks per hour plotted as function of day with Day 0 corresponding to the last day of the previous stage (pre-task baseline, when all bottles still contained plain water) (ANOVA: day F3,72=21.34 p <.0001 ω²=.18, sex F1,24=1.236 ns). Lick frequency increased instantly in response to the introduction of saccharin at the task sides. There was no evidence for a sex effect on lick frequency, suggesting that male and female mice consumed similar amounts of liquid. [file Image_1.TIFF]

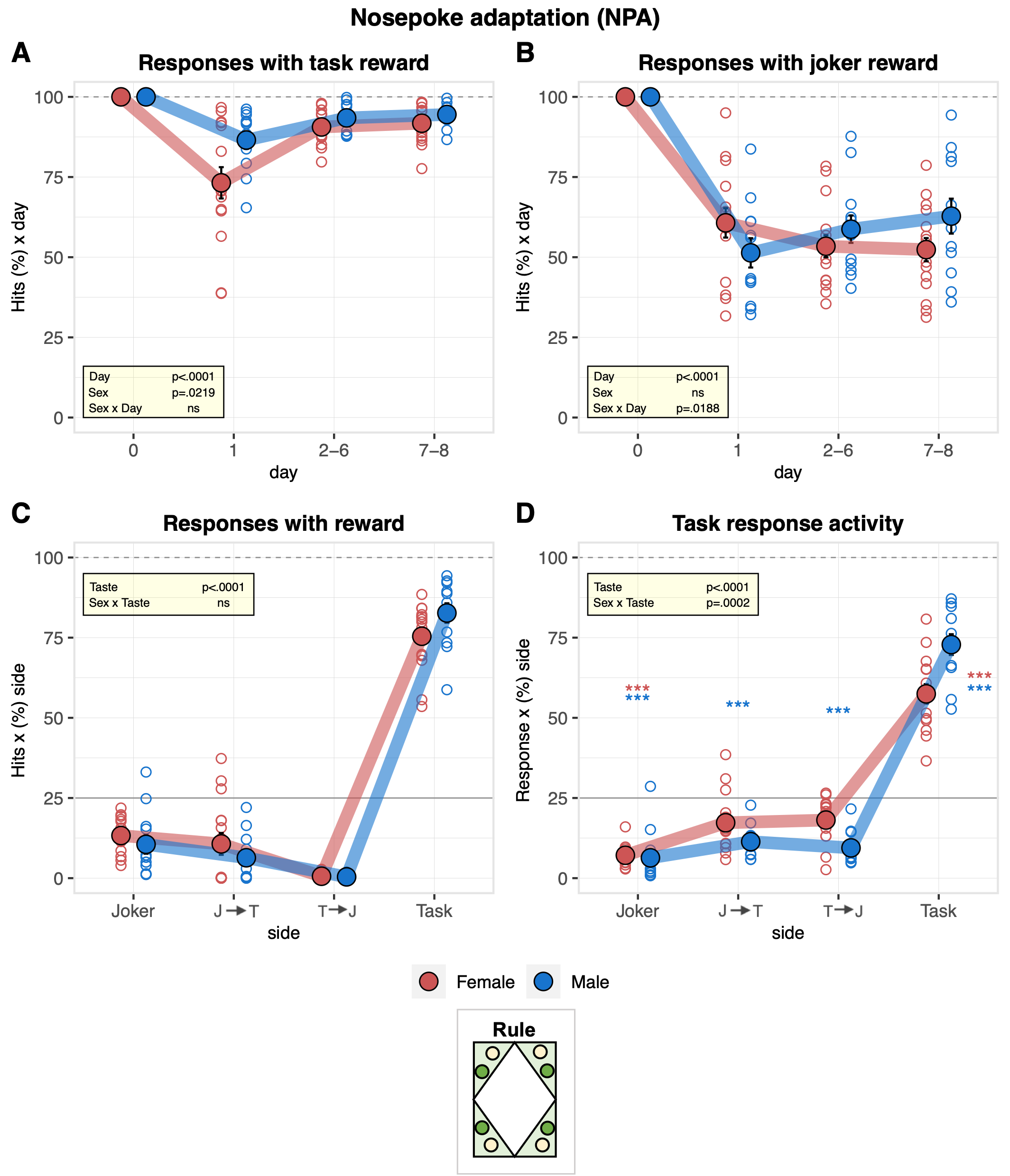

Supplement: SUPPLEMENTARY FIGURE S2 — Activity of male and female mice during the nosepoke adaptation stage. One-sample t-test results shown for groups separately in red and blue when a significant interaction with sex is present: ***p<.001 **p<.01 *p<.05. A) Percentage of nosepokes overlapping with accessibility of saccharin bottles plotted as function of day (ANOVA: day F3,69=143.9 p<.0001 ω²=.77, sex F1,23=6.040 p=.0219 ω²=.17, sex x day F3,69=2.571 ns, sex x choice F1,23=1.802 ns, Box-Cox λ 4.00). Percentage of nosepokes overlapping with accessibility of saccharin bottles dropped more strongly in females but sexes became indistinguishable by the end of the stage. B) Percentage of responses with nosepokes overlapping with accessibility of water bottles plotted as function of day (ANOVA: day F3,69=57.08 p<.0001 ω²=.58, sex F1,23=.3964 ns, sex x day F3,69=3.550 p=.0188 ω²=.06, sex x choice F1,23=1.110 ns, Box-Cox λ .500). Percentage of responses with nosepokes overlapping with accessibility of water bottles dropped more strongly in males but soon recovered to levels slightly higher than females. C) Percentage of responses with nosepokes overlapping with accessibility of water and saccharin reward plotted as function of task sequence during the visit (joker side only, joker side followed by task side, task side followed by joker side, task side only) and averaged across the entire stage (ANOVA: sex x taste F3,69=2.546 ns, sex x choice x taste F3,69=2.560 ns, Box-Cox λ .500). D) Percentage of responses plotted as function of side sequence during the visit (joker side only, joker side followed by task side, task side followed by joker side, task side only) and averaged across the entire stage (ANOVA: sex x taste F3,69=7.355 p=.0002 ω²=.20, sex x choice x taste F3,69=.9204 ns, Box-Cox λ .500). Males more strongly avoided to respond at both sides than females and showed a stronger preference for responding exclusively at the task side. [file Image_2.TIFF]

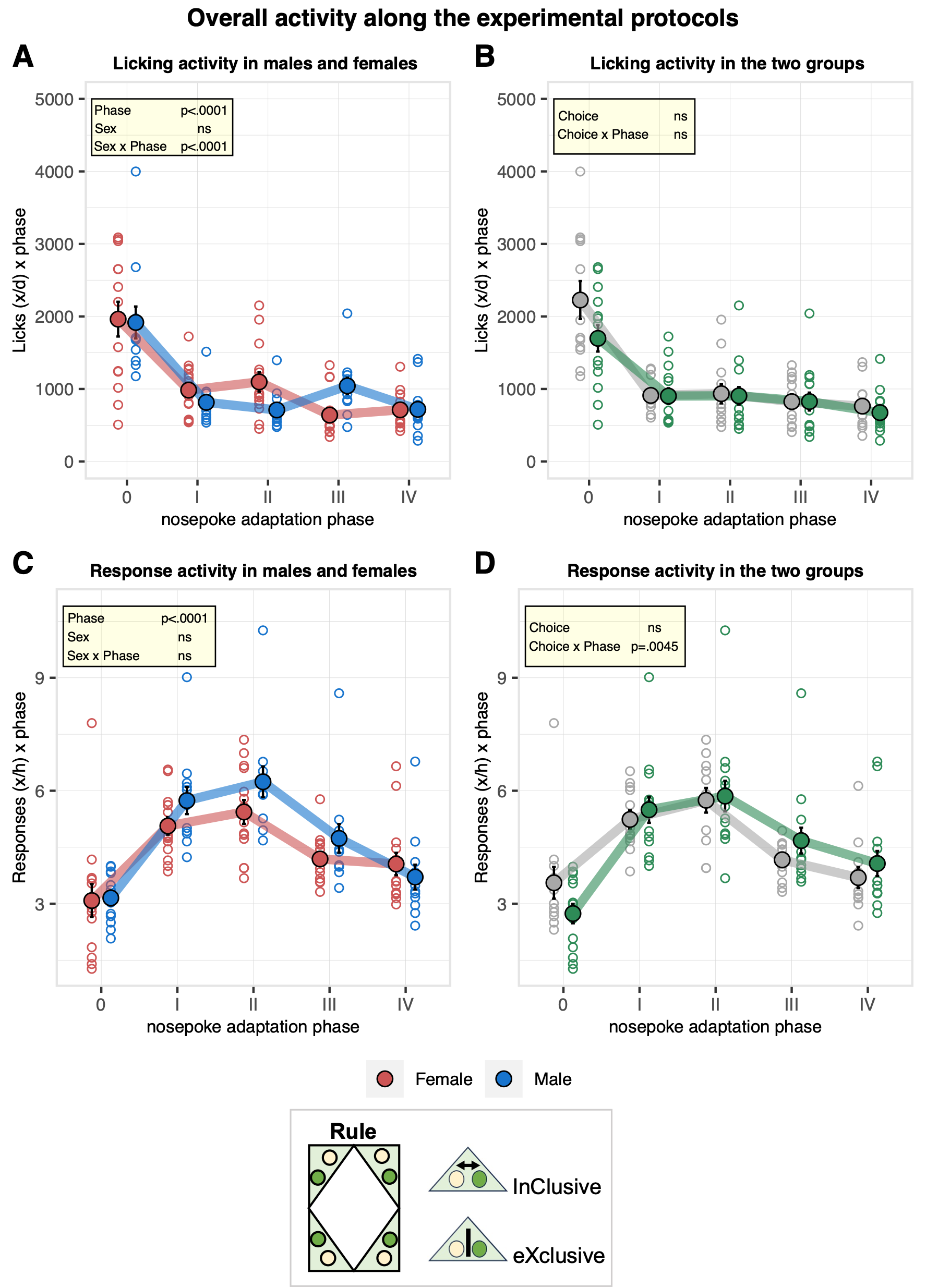

Supplement: SUPPLEMENTARY FIGURE S3 — Activity of the mice during nosepoke adaptation stages before, between and after learning tasks. A, B) Average licks per day plotted as function of phase each corresponding to the last 2 days of a stage (0 free adaptation saccharin / water, I first nosepoke adaptation, II continued pre-learning nosepoke adaptation, III nosepoke adaptation after corner preference training, IV final nosepoke adaptation after time place training; ANOVA: phase F4,88=46.46 p<.0001 ω²=.47, Box-Cox λ .500). At the transition from free to nosepoke adaptation, lick numbers dropped by about 50% and remained stable thereafter with only very small decreases after learning stages, without evidence for a consistent effect of sex on lick numbers. No evidence for a choice group effect on the overall lick number was observed. C, D) Average responses per hour plotted as function of phase (ANOVA: phase F4,88=60.69 p<.0001 ω²=.50, Box-Cox λ .500). Overall, responses increased strongly at the transition from free to nosepoke adaptation to decrease again after the learning stages without statistical evidence for a sex effect. Responses were also similar in the two experimental groups. [file Image_3.TIFF]

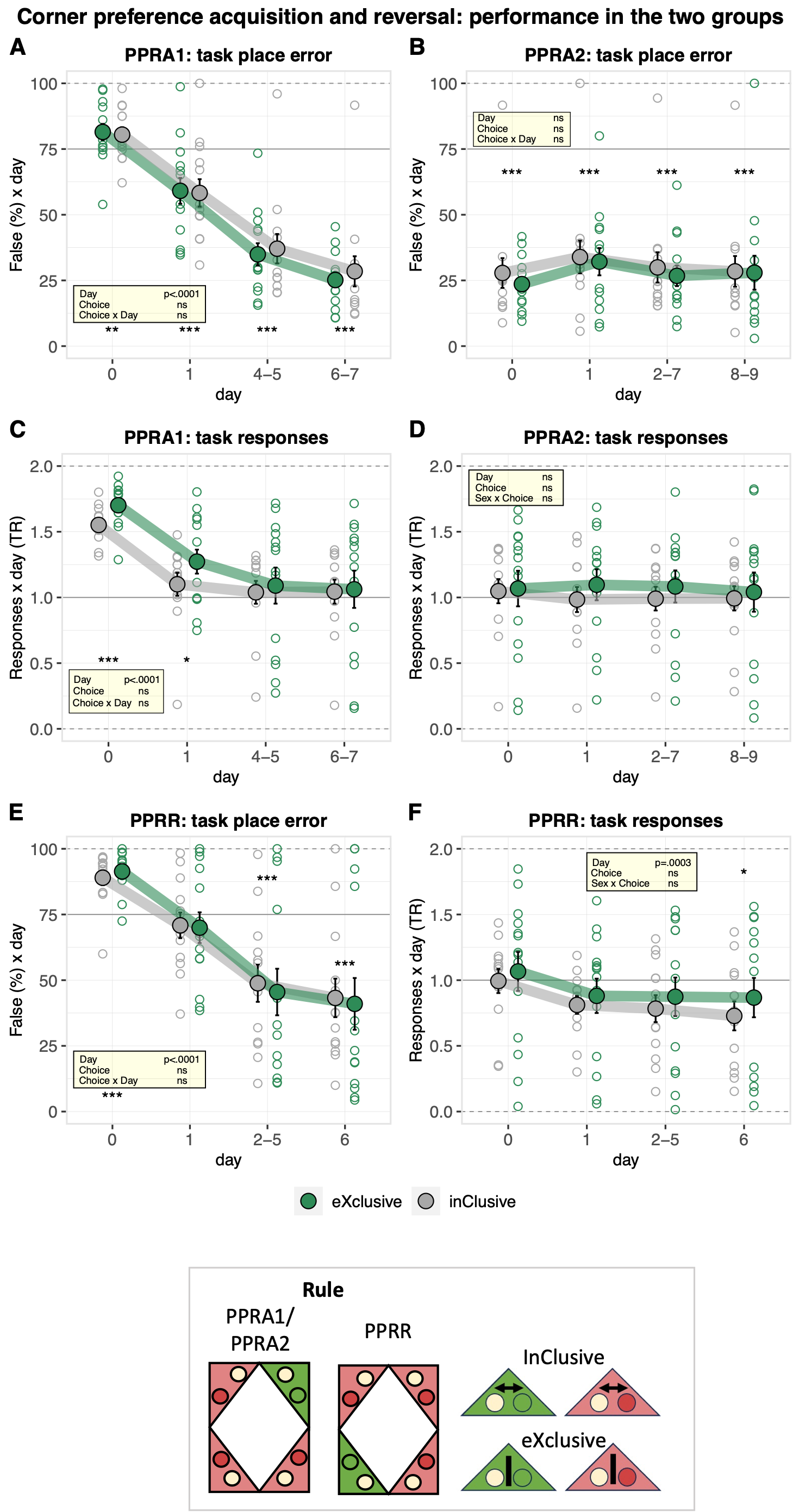

Supplement: SUPPLEMENTARY FIGURE S4 — Learning performance of the inclusive and exclusive groups in the corner preference acquisition and reversal stages. During corner preference acquisition 1 (PPRA1, 7 days), water was available at joker sides in all corners, but saccharin could only be obtained at the task side of a single target corner which remained the same throughout the task. Corner preference acquisition 2 (PPRA2, 9 days) continued with same target corner as in corner preference acquisition 1 after cleaning the cages. During corner preference reversal (PPRR, 6 days) the target corner was defined as opposite to the one used in corner preference acquisition 1 and 2. Ratio defined as (b + 2 x Task) / Proofs_Legends (b + Joker + Task), b=2, chance = 1, range = 0-2. One-sample t-tests against chance (solid horizontal line): ***p<.001 **p<.01 *p<.05 referring to the comparison of pooled groups against chance. A) Percentage of place errors in PPRA1 corresponding to task responses to incorrect corners plotted as function of day with Day 0 corresponding to the last 2 days of joker adaptation with saccharin still available in all corners (ANOVA: choice F1,23=.0326 ns). B) Percentage of place errors in PPRA2 corresponding to task responses to incorrect corners plotted as function of day with Day 0 corresponding to the last 2 days of corner preference acquisition 1 (ANOVA: choice F1,23=.1233 ns, Box-Cox λ .500). C) Response task ratio plotted as function of day in PPRA1 with Day 0 corresponding to the last 2 days of joker adaptation with saccharin still available in all corners. (ANOVA: choice F1,23=2.277 ns, Box-Cox λ 3.00). D) Response task ratio plotted as function of day in PPRA2 with Day 0 corresponding to the last 2 days of corner preference acquisition 1 (ANOVA: choice F1,23=.4011 ns, Box-Cox λ 2.00). E) Percentage of place errors during PPRR corresponding to task responses to incorrect corners plotted as function of day with Day 0 corresponding to the last 2 days of corner preference acquisition 2 [file Image_4.TIFF]

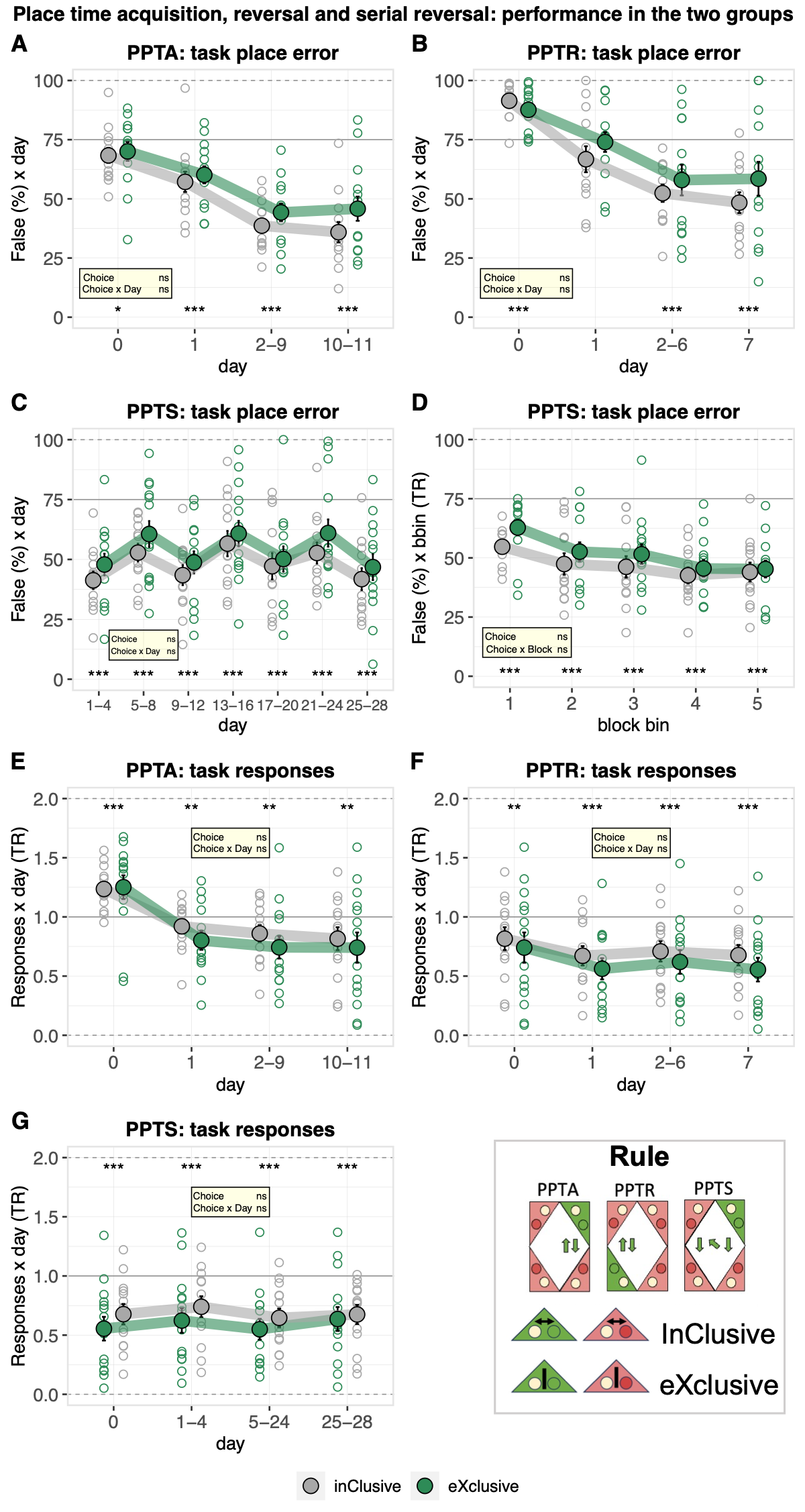

Supplement: SUPPLEMENTARY FIGURE S5 — Learning performance of the inclusive and exclusive groups in the place time acquisition, reversal and serial reversal stages. During place time acquisition (PPTA, 11 days), the target corner moved to the right at 14:00 every day and back to the original position at 02:00. During place time reversal (PPTR, 7 days), a new corner pair was defined with target corner opposite to the one used in time place acquisition and again moving to the right at 14:00 every day and back to the original position at 02:00. During place time serial reversal (PPTS, 28 days), 7 alternations between place time acquisition and reversal each lasting for 4 days, starting and ending with acquisition, were defined. One-sample t-tests against chance (solid horizontal line): ***p<.001 **p<.01 *p<.05 referring to the comparison of pooled groups against chance. A) Percentage of place errors during PPTA corresponding to task responses to incorrect corners plotted as function of day with Day 0 corresponding to the last 2 days of joker adaptation III with saccharin available in all corners (ANOVA: choice F1,23=2.463 ns). B) Percentage of place errors during PPTR corresponding to task responses to incorrect corners plotted as function of day with Day 0 corresponding to the last 2 days of time place acquisition (ANOVA: choice F1,23=.7869 ns). C) Percentage of place errors corresponding to task responses to incorrect corners plotted as function of day (7x4 days, average of each alternation) during PPTS (ANOVA: choice F1,23=2.106 ns). D) Percentage of place errors corresponding to task responses to incorrect corners plotted as function of block bin (first, second, third, fourth and fifth 20% responses in each alternation, averaged across alternations) during PPTS (ANOVA: choice F1,23=1.516 ns). E) Response task ratio plotted as function of day during PPTA with Day 0 corresponding to the last 2 days of joker adaptation III with saccharin available in all corners. Ratio defined as (2 + 2 x Task) / (2 + Jok [file Image_5.TIFF]
